# Gene Control during Transcription Elongation

**DOI:** 10.1155/2012/758384

**Published:** 2012-03-25

**Authors:** Sebastián Chávez, David S. Gross, Damien Hermand, Carlos Suñé

**Affiliations:** ^1^Departamento de Genética, Facultad de Biología, Universidad de Sevilla, Seville 41012, Spain; ^2^Department of Biochemistry and Molecular Biology, Louisiana State University Health Sciences Center, Shreveport, LA 71130-3932, USA; ^3^Laboratoire de Genetique Moleculaire, Academie Universitaire Louvain, 5000 Namur, Belgium; ^4^Departamento de Biología Molecular, Instituto de Parasitología y Biomedicina “López Neyra” (IPBLN-CSIC), Parque Tecnológico Ciencias de la Salud, 18100 Armilla, Spain

This special issue of Genetics Research International is dedicated to transcription elongation and the role that it plays in the control of gene expression. As K. Brannan and D. Bentley highlight in their retrospective review, 30 years after the first examples of gene control during transcription elongation were described, the importance of this step of the RNA biogenesis process has become increasingly clear, in parallel with myriad findings that connect transcription elongation to almost every relevant genome-related phenomenon. It is, therefore, gratifying to present the most current work from an array of leading scientists, who offer in one rich volume up-to-date review articles of this interesting field.

Addressed are the covalent modifications of the C-terminal domain of the largest subunit of RNA polymerase II, whose extensive studies have launched the interesting paradigm of a CTD code governing and integrating cotranscriptionally the different steps of mRNA biogenesis. The teams led by A. Greenleaf (B. Bartkowiak et al.) and A. Ansari (D. Zhang et al.) review this important aspect of RNA-polymerase-II-dependent transcription. They discuss the readers, writers, and erasers of the posttranslational modifications that occur on the CTD and that function as a binding platform for enzymatic complexes that regulate Pol II elongation, pre-mRNA splicing, RNA export from the nucleus, chromatin remodelling, and DNA repair.

Also featured is the way transcription is ruled by the other great code, that is, the histone code, a panoply of specific interactions between genome effectors and chromatin that is mediated by covalent modifications of the histones. E. M. Crisucci and K. M. Arndt explore the roles of the Paf1 complex (Paf1C) in regulating gene expression in both budding yeast and metazoans. They review evidence that Paf1C associates with elongating Pol II, and by doing so facilitates histone modifications to the underlying chromatin template as well as contributes to transcription termination and RNA 3′-end formation. A complementary view of chromatin dynamics during transcription elongation is provided by A. A. Duina, who provides a comprehensive review of the roles of the histone chaperones Spt6 and FACT in facilitating passage of Pol II on the chromatin template. He compares and contrasts their mechanisms, highlighting recent evidence indicating that they travel across transcribed regions in likely association with elongating Pol II, where they play an important role in the removal and redeposition of nucleosomes during polymerase elongation. Cotraversal of Pol II and its regulatory factors is also an important aspect of those regulatory phenomena affecting the transition between initiation and elongation and during early elongation. L. A. Stargell and colleagues (M. N. Yearling et al.) describe the complex interplay among the constellation of factors that govern the transition of poised RNA polymerases into active elongation.

The posttranscriptional fate of RNA is paradoxically dictated cotranscriptionally. mRNA export and alternative splicing are coupled to transcription elongation as the teams led by A. Kornblihtt (M. de la Mata et al.) and S. Rodriguez-Navarro (M. M. Molina-Navarro et al.) explain in their reviews. C. Suñé and colleagues (M. Sánchez-Álvarez et al.) show us that this coupling is not only temporal but also spatial and describe the increasing progress of light microscopy at single-cell resolution to characterize transcription factories and interchromatin granule clusters within the nucleus.

mRNAs are not the only RNA species produced by RNA polymerase II. J. Colin et al. guide us through the fascinating world of cryptic transcripts and their connection to the control of gene expression by early transcription termination. The coexistence of canonical and cryptic transcription is a challenge for genomicists when addressing global transcription. J. E. Pérez-Ortín and colleagues address this and other problems in their review on how to measure the different steps of gene expression genomewide.

Control of transcription elongation is essential for understanding the regulatory behaviour of the genome; accordingly, the number of genes known to be regulated at the elongation level is exponentially increasing in the literature. HIV transcription is a classic model of gene regulation during elongation. K. A. Nilson and D. H. Price review it, with special attention to the roles of the viral Tat protein, the best characterized gene-specific effector of transcription elongation, and with a view on the therapeutic approaches to AIDS that targets HIV transcription. As E. de Nadal and F. Posas describe, stress-responsive genes provide another good example where transcription elongation is highly regulated. Moreover, functional contributions of transcription elongation reach fields beyond gene expression, like V(D)J recombination during the generation of antigen receptor diversity in T and B lymphocytes. The team of C. Hernández-Munain (B. del Blanco et al.) explains in their review how the elongation activity of RNA polymerase II is required for creating accessible chromatin for the RAG1 and RAG2 recombinases to initiate recombination.

Transcription elongation by RNA polymerase II is the focus of most reviews in this special issue, but elongation by other RNA polymerases is also attracting the interest of the scientific community. This is the case for O. Gadal and his colleagues (B. Albert et al.), who describe what is known of the role of RNA polymerase I elongation in regulating rRNA production.

Overall, the articles in this special issue describe the present state of transcription elongation. Ongoing advances in this field will certainly propel our capacity to more deeply understand gene expression and genome dynamics. In this regard, these reviews all provide a perspective into the future of gene control during transcription elongation.

*Sebastián Chávez* *Sebastián Chávez* *David S. Gross* *David S. Gross* *Damien Hermand* *Damien Hermand* *Carlos Suñé* *Carlos Suñé*

